# Supplementary material for: Genetic Dissection of Growth and Eco-Physiological Traits Associated with Altitudinal Adaptation in Sakhalin Fir (Abies sachalinensis) Based on QTL Mapping
Source: Genes (Basel). 2021 Jul 22;12(8):1110. doi: 10.3390/genes12081110 (PMC8392833; doi:10.3390/genes12081110)
Supplement: Supplementary file 1 [file genes-12-01110-s001.zip › genes-1284396-supplementary.pdf]

Supplementary Materials S1

Supplementary Materials S1.1. Protocol of ddRAD-seq

250 ng of DNA template from each individual was double-digested with SphI-HF (10 units per reaction, New England Biolabs Inc., Beverly, MA, USA) and PstI (10 units per reaction, New England Biolabs Inc.) at 37 °C for 16 h. Each fragmented sample was purified using the homemade AMPure beads (using Sera-Mag SpeedBeads (Thermo Scientific, Waltham, MA, USA), hereafter, AMPure beads), eluted with 25 μL of low Te buffer (pH 8.0). Purified samples were then ligated to CS1-tagged and CS2-tagged adapters, consisting of the common sequence tag 1 (CS1) and the common sequence tag 2 (CS2) of Access Array, respectively. The CS1-tagged adapter binds to overhangs generated by PstI, and the CS2-tagged adapter contains overhangs compatible with an SphI site. Adapter ligation was performed in a 40 μL reaction volume containing 25 μL of DNA samples, 4 μL of CS1-tagged adapter (100 nM), 4 μL of CS2-tagged adapter (100 nM), 1 μL of T4 ligase (1 U/μL, Invitrogen, Carlsbad, CA, USA) and 8 μL of 5 × T4 ligation buffer (Invitrogen, Carlsbad, California, USA). The reaction was incubated at 25 °C for 1 h, and the ligase was then heat inactivated at 65 °C for 30 min. After adapter ligation, each DNA samples was purified using AMPure beads and amplified using KAPA HiFi polymerase (KAPA biosystems, Woburn, MA, USA) with 400 nM primers of Access Array Barcode Library for Illumina Sequencer-384 (Fluidigm Corp., South San Francisco, CA, USA). The following PCR protocol was used: initial denaturation at 95 °C for 5 min; 12 cycles of 95 °C for 15 s, 60 °C for 30 s and 72 °C for 1 min; followed by a final extension period at 72 °C for 3 min. The amplified libraries were purified using AMPure beads and quantified using the Qubit fluorometer with a Quant-it dsDNA HS kit (Invitrogen). The equal amount of DNA from each sample was mixed and size-selected using the BluePippin agarose gel (2% agarose cartridge, Sage Science, Beverly, MA, USA) under a "narrow" setting with a mean of 450 bp. After size-selection, each sample was purified using AMPure beads. The quality, size and concentration of the pooled libraries were finally determined using the 2100 Bioanalyzer with a high sensitivity DNA chip (Agilent technologies, Waldbronn, Germany), and the library was diluted before template preparation.

Supplementary Materials S1.2. Sequence of #1970 Loci Derived from ddRAD-seq

GTCCATTATACACAAATTGATACAAACAATATTTCAAATTGGT
GAAATGCCTACAGGTTTTTAAAAACCCCCAAAATCAAGAAAC
TAATTATCAAGATTCNNNNNNNNNNTCGATGACTTTTCGATC
TATAATACTGTCATCCACAGCCAGCACATGAAGCTGGGAAGTG
CTCTCTCCCTCTACGCCGCTCAATTGATCTGAAGTCGAAGGCAT
GC

Supplementary Materials S2

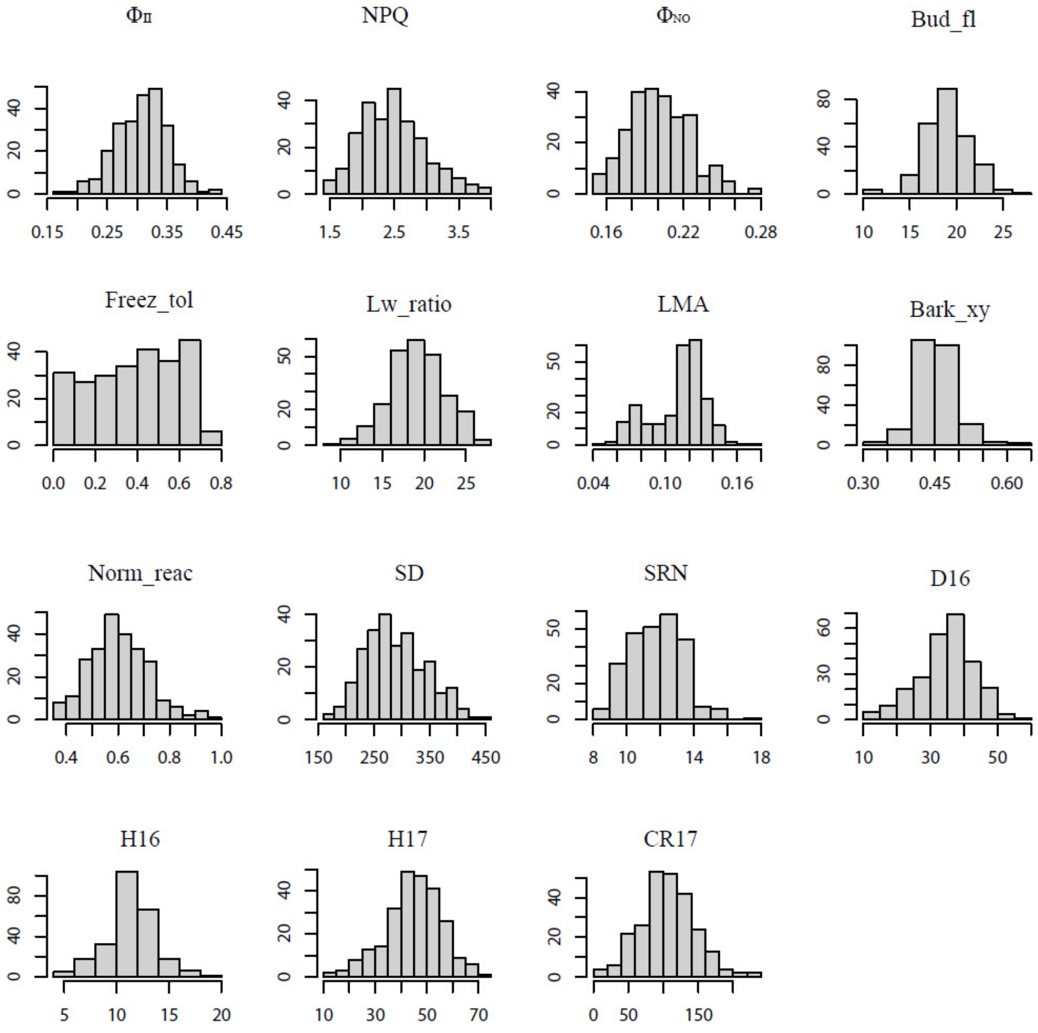

Figure S1. Histograms of the functional traits measured in the present study.

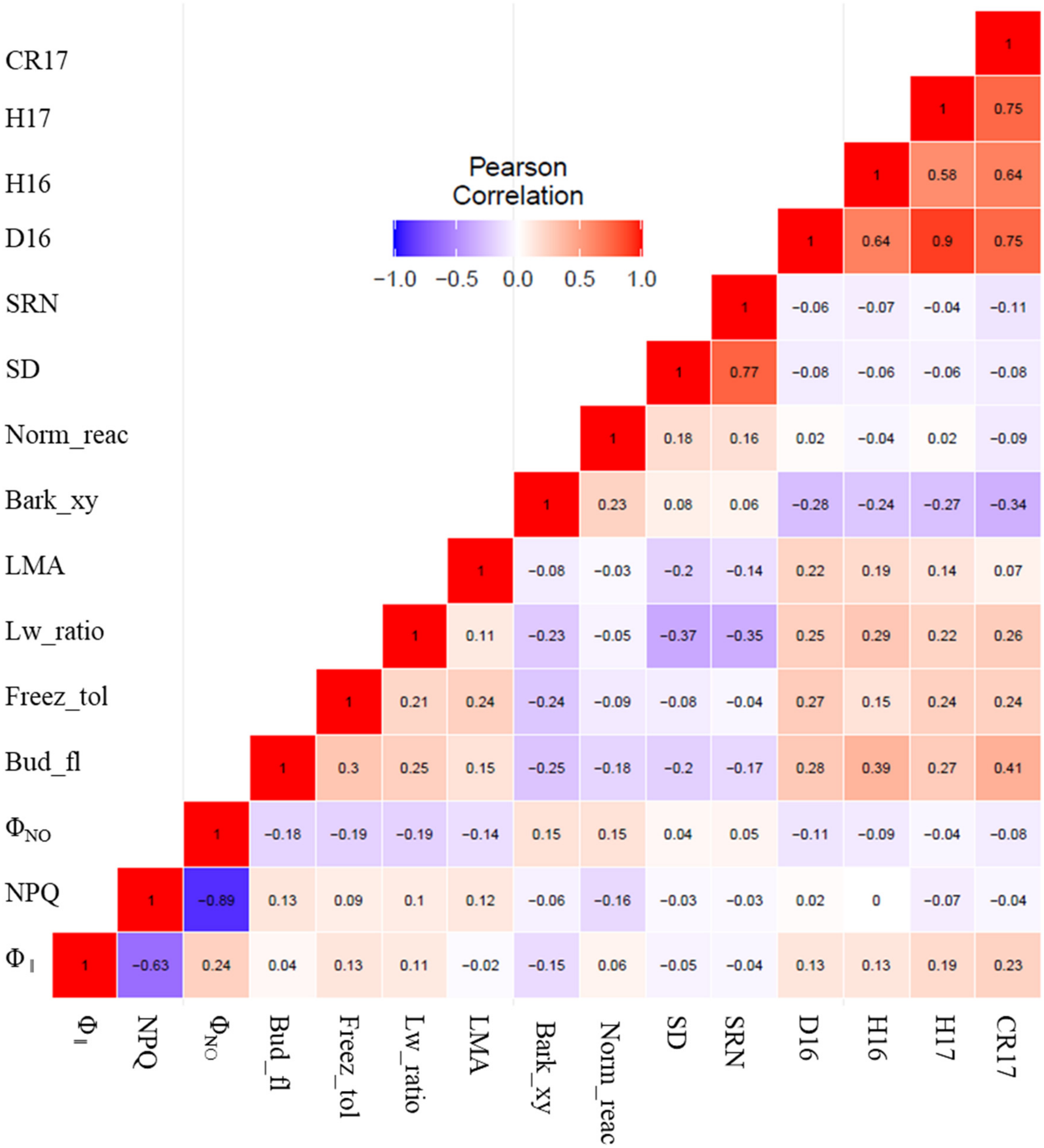

Figure S2. Correlations among functional traits.

Table S1. Model comparison in case of *p*0 = 1, 3, 5, 7 and 9 [62]. * elpd-se > 0, ** elpd-se > se-diff, other traits were not significant between the null model and the full model.

Trait	Parameter	*p*0 (1)		*p*0 (3)		*p*0 (5)		*p*0 (7)		*p*0 (9)	
NPQ	elpd_diff	−1.57		−2.84		−3.18		−3.53		−3.91	
	se_diff	1.36		2.13		2.28		2.42		2.59	
	elpd-se	0.22	*	0.72	*	0.89	*	1.11	*	1.32	*
Φ_{NO}	elpd_diff	−14.6		−14.8		−14.9		−15.4		−15.5	
	se_diff	5.30		5.39		5.39		5.53		5.62	
	elpd-se	9.28	**	9.41	**	9.54	**	9.88	**	9.90	**
Bud_fl	elpd_diff	−1.14		−2.50		−3.42		−3.83		−3.48	
	se_diff	1.40		2.19		2.54		2.64		2.70	
	elpd-se	−0.26		0.31	*	0.88	*	1.20	*	0.79	*
Freez_tol	elpd_diff	−2.98		−4.71		−5.64		−5.35		−5.57	
	se_diff	2.41		3.11		3.43		3.44		3.55	
	elpd-se	0.56	*	1.61	*	2.21	*	1.91	*	2.03	*
Lw_ratio	elpd_diff	−5.54		−7.01		−7.85		−8.57		−8.10	
	se_diff	3.17		3.70		3.98		4.15		4.10	
	elpd-se	2.37	*	3.30	*	3.87	*	4.41	**	3.99	*
LMA	elpd_diff	−0.30		−0.41		−0.46		−0.57		−0.64	
	se_diff	0.22		0.37		0.41		0.56		0.60	
	elpd-se	0.08	*	0.05	*	0.05	*	0.01	*	0.05	*
D16	elpd_diff	−1.86		−3.15		−3.69		−3.21		−3.56	
	se_diff	1.61		2.26		2.57		2.51		2.65	
	elpd-se	0.25	*	0.89	*	1.13	*	0.70	*	0.91	*
H16	elpd_diff	−11.6		−12.4		−13.0		−12.7		−13.2	
	se_diff	4.25		4.44		4.61		4.57		4.69	
	elpd-se	7.34	**	7.98	**	8.40	**	8.17	**	8.53	**
H17	elpd_diff	−6.2		−8.24		−9.22		−9.12		−9.22	
	se_diff	3.16		3.78		3.96		3.96		4.10	
	elpd-se	3.04	*	4.46	*	5.26	**	5.15	**	5.12	**
CR17	elpd_diff	−13.4		−14.3		−14.6		−15.6		−15.8	
	se_diff	4.55		4.73		4.80		5.01		5.06	
	elpd-se	8.83	**	9.56	**	9.83	**	10.6	**	10.7	**

Table S2. Description of candidate genes underlying QTLs in the present study.

Locus ID	Transcript ID	Description	Gene Ontology	KEGG Orthology
#1970	AbisacEGm029091t1	response regulator 3	GO:0000160 BP phosphorelay signal transduction system	K14492 ARR-A; two-component response regulator ARR-A family
#2055	AbisacEGm065081t1	S-adenosyl-L-methionine-dependent methyltransferases superfamily protein	-	-
#6787	AbisacEGm003877t1	protein transport, Golgi organization	-	-
#7510	AbisacEGm055724t1	DNA-directed DNA polymerases	-	-
#10164	AbisacEGm041262t1	cytochrome P450, family 71, subfamily A, polypeptide 23	-	-
#12865	AbisacEGm017785t6	AGC (cAMP-dependent, cGMP-dependent and protein kinase C) kinase family protein	-	-
#13364	AbisacEGm065825t1	timeless family protein	-	K03155 TIMELESS; timeless
#25432	AbisacEGm017785t5	homocysteine methyltransferase 2	-	-
